# Platelet-Rich Plasma Increases the Levels of Catabolic Molecules and Cellular Dedifferentiation in the Meniscus of a Rabbit Model

**DOI:** 10.3390/ijms17010120

**Published:** 2016-01-16

**Authors:** Hye-Rim Lee, Oog-Jin Shon, Se-Il Park, Han-Jun Kim, Sukyoung Kim, Myun-Whan Ahn, Sun Hee Do

**Affiliations:** 1 Department of Veterinary Clinical Pathology, College of Veterinary Medicine, Konkuk University, Seoul 143-701, Korea; monade23@hanmail.net (H.-R.L.); vet.hanjun@gmail.com (H.-J.K.); 2 Department of Orthopedic Surgery, College of Medicine, Yeungnam University, Daegu 705-717, Korea; ossoj@med.yu.ac.kr (O.-J.S.); mwahn@ynu.ac.kr (M.-W.A.); 3 Cardiovascular Product Evaluation Center, College of Medicine, Yonsei University, Seoul 120-752, Korea; SAILING@yuhs.ac; 4 School of Materials Science and Engineering, Yeungnam University, Gyeongsan 712-749, Korea; sykim@ynu.ac.kr

**Keywords:** platelet-rich plasma, meniscal cells, MMPs, proteoglycan, collagen

## Abstract

Despite the susceptibility to frequent intrinsic and extrinsic injuries, especially in the inner zone, the meniscus does not heal spontaneously owing to its poor vascularity. In this study, the effect of platelet-rich plasma (PRP), containing various growth factors, on meniscal mechanisms was examined under normal and post-traumatic inflammatory conditions. Isolated primary meniscal cells of New Zealand white (NZW) rabbits were incubated for 3, 10, 14 and 21 days with PRP(−), 10% PRP (PRP(+)), IL(+) or IL(+)PRP(+). The meniscal cells were collected and examined using reverse-transcription polymerase chain reaction (RT-PCR). Culture media were examined by immunoblot analyses for matrix metalloproteinases (MMP) catabolic molecules. PRP containing growth factors improved the cellular viability of meniscal cells in a concentration-dependent manner at Days 1, 4 and 7. However, based on RT-PCR, meniscal cells demonstrated dedifferentiation, along with an increase in type I collagen in the PRP(+) and in IL(+)PRP(+). In PRP(+), the aggrecan expression levels were lower than in the PRP(−) until Day 21. The protein levels of MMP-1 and MMP-3 were higher in each PRP group, *i.e.*, PRP(+) and IL(+)PRP(+), at each culture time. A reproducible 2-mm circular defect on the meniscus of NZW rabbit was used to implant fibrin glue (control) or PRP *in vivo*. After eight weeks, the lesions in the control and PRP groups were occupied with fibrous tissue, but not with meniscal cells. This study shows that PRP treatment of the meniscus results in an increase of catabolic molecules, especially those related to IL-1α-induced inflammation, and that PRP treatment for an *in vivo* meniscus injury accelerates fibrosis, instead of meniscal cartilage.

## 1. Introduction

Various cartilaginous tissues such as articular cartilage, menisci, and other fibrocartilages, differ from each other with respect to molecular composition [1,2]. The unique functional properties of each type of cartilage depend mainly on the composition of the extracellular matrix (ECM), such as collagens and proteoglycans [3]. The meniscus is a wedge-shaped semi-lunar disc that plays an important role in knee function. Meniscal tissue contains mainly water (72%), collagens (22%), and glycosaminoglycans (0.8%) [4]. Type I collagen accounts for over 90% of the total collagen content. Types II–V collagen are the remaining meniscal collagens [4,5]. The peripheral two-thirds of the meniscus consists of type I collagen, whereas type II collagen comprises a significant portion of the inner meniscus with chondrocyte-like cells [4,5,6,7]. One of the interesting features of meniscus healing arises from the finding that the tissue is differentially vascularized, with fibrocartilaginous tissue in the vascularized region (red outer zone) and cartilaginous tissue in the avascular region (white inner zone). The inner zone lacks the innate ability for self-repair thus, damage to the meniscus can be permanent, leading to a loss of function [8,9]. Maintenance of the meniscal cartilage is highly important for preventing the accelerated degeneration of the knee joint [10,11], which means we need to understand the cellular mechanisms responsible for meniscal degradation and the cellular matrix changes necessary for intrinsic or therapeutic meniscal repair.

Meniscus tears and degeneration are common, and natural healing is limited [12,13,14]. Pathological changes in the meniscus can be caused by direct injury or joint inflammation, and contribute to the initiation and progression of synovitis and osteoarthritis (OA) of the knee joint. The pro-inflammatory cytokines interleukin-1 (IL-1) and tumor necrosis factor-alpha (TNF-α) are upregulated in injured joints as well as the meniscus [7,12,13,14,15]. In turn, these increased levels of IL-1α can suppress matrix biosynthesis and accelerate enzymatic degradation in the affected lesion. Specifically, IL-1α increases the expression and activity of matrix metalloproteinases (MMPs) in meniscal cells and explants [16,17]. Meniscal cells express MMP-1, MMP-2, MMP-3, MMP-8, MMP-9, and MMP-13 [18,19]. In patients with meniscal injuries, MMP-3 levels are elevated 30- to 40-fold, and levels of tissue inhibitor of metalloproteinases (TIMP)-1 increased 10-fold in the synovial fluid within 24 h of injury [20,21,22]. However, the role of MMPs and other biomechanical molecules in integrative meniscal repair is not fully understood, especially when platelet-rich plasma (PRP) is used as a growth factor source.

PRP is an autologous enriched source of various growth factors, including transforming growth factor-beta (TGF-β), vascular endothelial growth factor (VEGF), platelet-derived growth factor (PDGF), fibroblast growth factor (FGF), and insulin growth factor-1 (IGF-1) [22,23]. One of the most potent stimulators of matrix deposition in meniscal cells is TGF-β, although other growth factors such as FGF-2, PDGF, and IGF-1 can increase matrix production [24,25]. TGF-β and FGF-2 have been shown to increase meniscus cell proliferation in monolayer culture as well as in tissue regeneration [25]. In addition, TGF-β, IGF-1 and PDGF support the activity of meniscal cells [24,25,26]. Based on the positive effects of PRP on cell proliferation, collagen synthesis, and vascularization, PRP has been used as a potential biological stimulus for meniscus tissue regeneration [25,26,27,28,29].

Previous studies have described the regenerative effects of PRP and several growth factors, especially PDGF and TGF-β, for meniscal repair by *in vivo* [19,25,30,31,32]. However, most previous studies have not used PRP itself, but a PRP/hydrogel composite scaffold. Additionally, previous studies have not reported the roles of each growth factor in meniscus injury. For the clinical application of growth factors, PRP is a strong candidate resource owing to its natural autologous growth factors [33,34,35,36,37]. In this study, we investigated the role of PRP in meniscal tissues and examined its role in the presence of IL-1, including the sequential and temporal modulation of mechanical functional proteins, such as aggrecan, type II collagen, MMPs, TGF-β, and PDGF, using meniscal cells and New Zealand white (NZW) rabbits.

## 2. Results

### 2.1. In Vitro

#### 2.1.1. Assessment of the Prepared PRP

**Figure 1:**
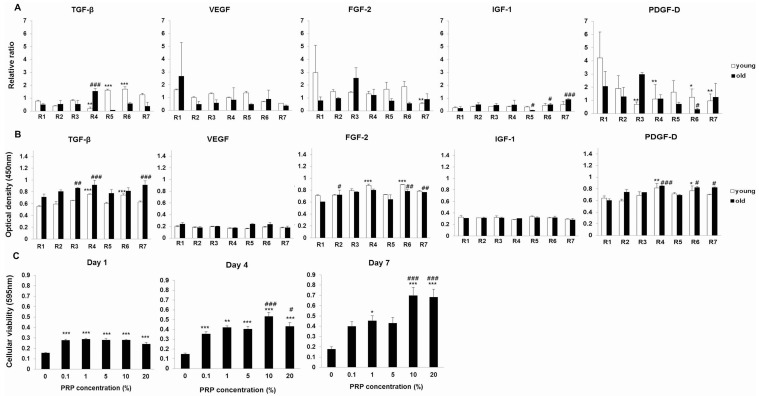
(**A**) Assessment of individual growth factors in platelet-rich plasma (PRP) by semi-quantitative RT-PCR. * *p* < 0.05, ** *p* < 0.01, *** *p* < 0.001 *versus* individual Y rabbits. ^#^ *p* < 0.05, ^###^ *p* < 0.001 *versus* individual O rabbits; (**B**) Evaluation of individual growth factors containing PRP from young rabbits (Y) and old rabbits (O) by ELISA. * *p* < 0.05; ** *p* < 0.01; *** *p* < 0.001 *versus* individual Y rabbits. ^#^ *p* < 0.05; ^##^ *p* < 0.01; ^###^ *p* < 0.001 *versus* individual O rabbits; (**C**) Cellular viability after PRP treatments. Each value is expressed as the mean ± standard error. The *p*-value is approximate (from a chi-square distribution). * *p* < 0.05, ** *p* < 0.01, *** *p* < 0.001 *versus* the untreated cells. ^#^ *p* < 0.05; ^###^ *p* < 0.001 *versus* the 0.1% PRP-treated cells.

In the analysis of growth factor expression in PRP samples, a mean platelet count of 4 × 10^6^ platelets/μL, using semi-quantitative reverse-transcription polymerase chain reaction (RT-PCR), revealed a negligible relationship between growth factor levels and both animal age and body weight of NZW rabbits (Figure 1A). The mean body weight was 1.5–1.7 kg for the young (Y, age 4 weeks) group and 2.3–2.6 kg for the old (O, age 16 weeks) group. Semi-quantitative RT-PCR revealed the major functional growth factors of PRP were most highly expressed in the Y group. Based on ELISA (enzyme-linked immunosorbent assay), the growth factor expression levels were higher in the O group than that in the Y group. Similar to previous results in humans, growth factors of NZW rabbits have no relationship with aging.

#### 2.1.2. Effects of PRP on Meniscal Cell Viability

A 3-(4,5-dimethylthiazol-2-yl)-2,5-diphenyl-tetrazolium bromide (MTT) viability assay revealed that PRP increased cellular viability and meniscal cells activity in a dose-dependent manner, except in the presence of 20% PRP (Figure 1C). The results were consistent with concentration- and time-dependent PRP-mediated promotion of meniscal cell viability.

#### 2.1.3. Effects of PRP on Gene and Protein Expression of Meniscal Cells

**Figure 2:**
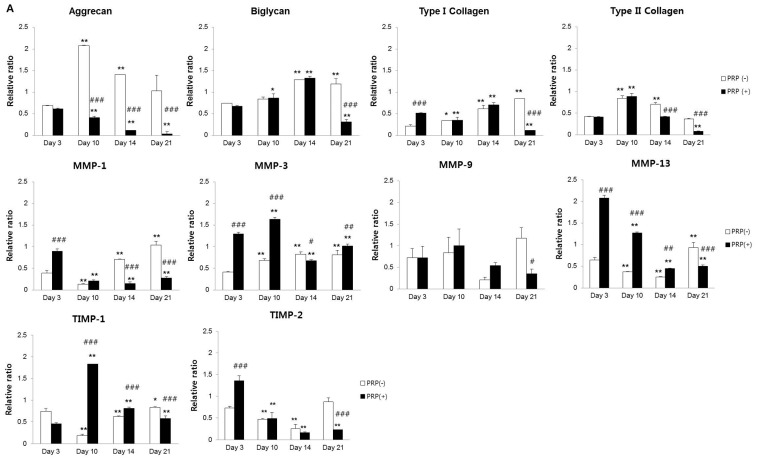

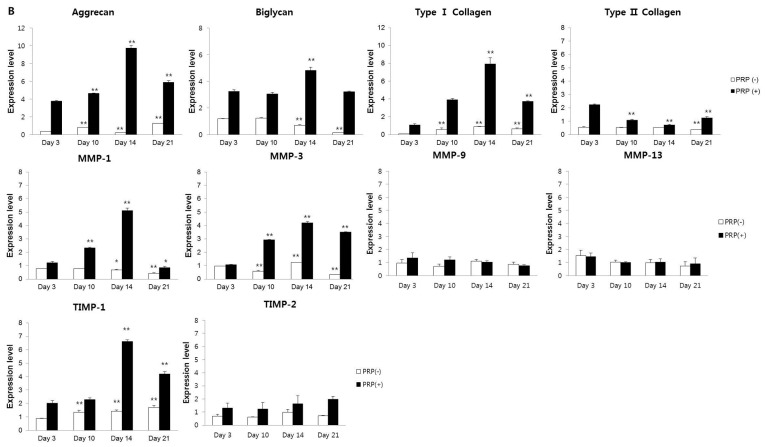
(**A**) mRNA expression in meniscal cells with/without PRP. Aggrecan mRNA expression decreased in a time-dependent manner in PRP(+). Type II collagen gene expression decreased in PRP(+) compared to PRP(−) after Day 10. In initial examinations, MMP-3, MMP-9, and MMP-13 displayed increased expression in the PRP(+); (**B**) Protein expression analysis in culture media of meniscal cells with/without PRP. PRP(+) showed a relative upregulation in the protein levels of MMP-1, MMP-3, and TIMP-1 compared to PRP(−). * *p* < 0.05; ** *p* < 0.01 *versus* Day 3. ^#^ *p* < 0.05; ^##^ *p* < 0.01; ^###^ *p* < 0.001 *versus* PRP(−).

The results of meniscal cell-related mRNA expression after culture with 10% PRP (PRP(+))are summarized Figure 2. PRP(+) a decreased in aggrecan over time. mRNA expression levels of type I collagen and biglycan were upregulated in both PRP(−) and PRP(+) up until Day 14 of the culture period. Type II collagen gene expression decreased sharply after Day 10 in PRP(+) compared to PRP(−) and, PRP(+) was reduced to low levels, by approximately 50%, at Day 14 compared to Day 10. PRP(+) exhibited decreased type II collagen and TIMP-1 expression after Day 10 (Figure 2A). The PRP(+) initially showed a relatively higher mRNA expression of MMP-1, MMP-3, and MMP-13 than did the PRP(−) at Days 3 and 10. MMP-13 and TIMP-2 expression decreased in a time-dependent manner in the PRP(+). Aggrecan expression in PRP(+) was very low during the same period. MMP-1, MMP-3, and TIMP-1 showed similar protein expression patterns to those observed at the mRNA level in PRP(+), which affected aggrecan, biglycan, and type II collagen expression. There were no significant differences in the levels of MMP-9 and MMP-13 protein expression in both PRP(−) and PRP(+) (Figure 2B).

Conclusively, in the PRP(+) group, RT-PCR showed decreased ECM content in meniscal cells and an immunoblot analysis revealed ECM degradation by MMP expression in culture media.

#### 2.1.4. Effects of IL-1α and PRP on Gene and Protein Expression in Meniscal Cells

**Figure 3:**
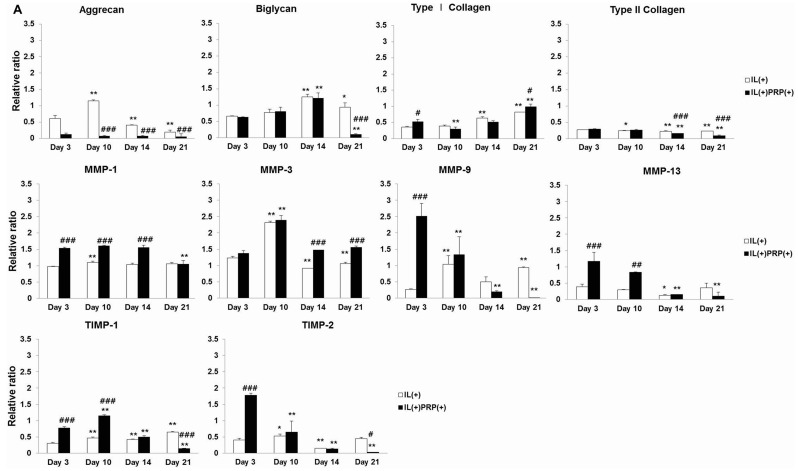

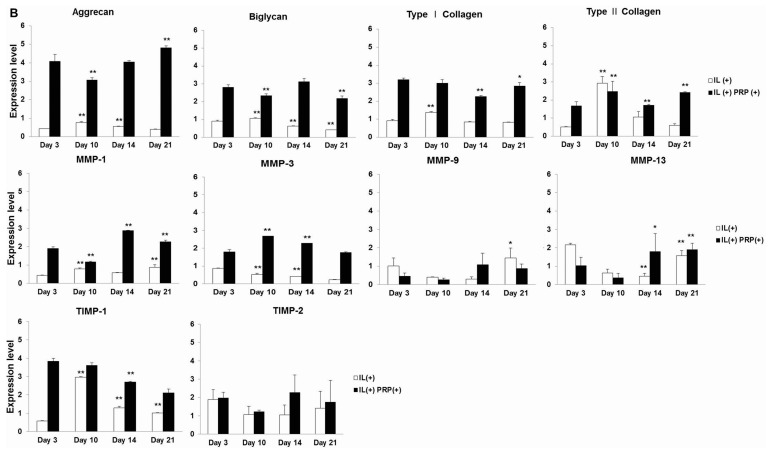
(**A**) mRNA expression in IL-1α-treated meniscal cells with/without PRP. mRNA expression of aggrecan and type II collagen was generally low compared to that observed in IL(+). The aggrecan and type II collagen expression levels were lower in IL(+)PRP(+) than IL(+); (**B**) Immunoblot analysis in IL-1α-treated culture media of meniscal cells with/without PRP. At every time point, protein expression levels of MMP-1, MMP-3, and TIMP-1 were higher in IL(+)PRP(+) than in IL(+), and there was no significant difference in their time courses. * *p* < 0.05; ** *p* < 0.01 *versus* Day 3. ^#^ *p* < 0.05; ^##^ *p* < 0.01; ^###^ *p* < 0.001 *versus* IL(+).

Treatment with the pro-inflammatory cytokine IL-1α induced meniscal cell injury and served as a surrogate for *in vivo* experiments. The relative mRNA expression for cartilage-specific proteins, especially aggrecan, in the IL(+)PRP(+) was lower than that in the IL(+) (Figure 3A). Biglycan expression showed a similar time-dependent pattern in the IL(+) and IL(+)PRP(+). The mRNA level of aggrecan decreased in the IL(+) from Day 10, whereas type I collagen gradually increased in the IL(+). High gene expression levels of MMP-1 were maintained until Day 14, and type II collagen levels were markedly decreased during the experimental period in the IL(+)PRP(+). IL(+)PRP(+) showed a time-dependent decrease in MMP-9, MMP-13, and TIMP-2 gene expression. There was a high level of type I collagen in IL(+)PRP(+) culture media.

#### 2.1.5. Temporal Sequential Modulation of PRP-Derived Growth Factors on Meniscal Cells

**Figure 4:**
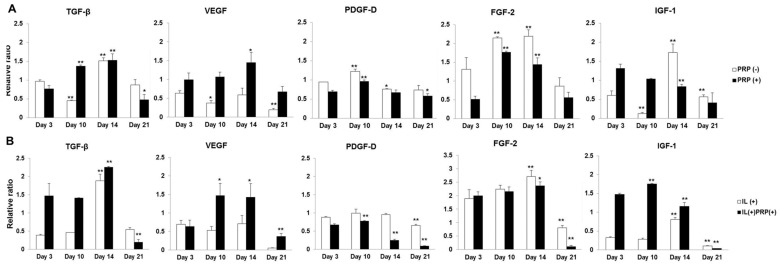

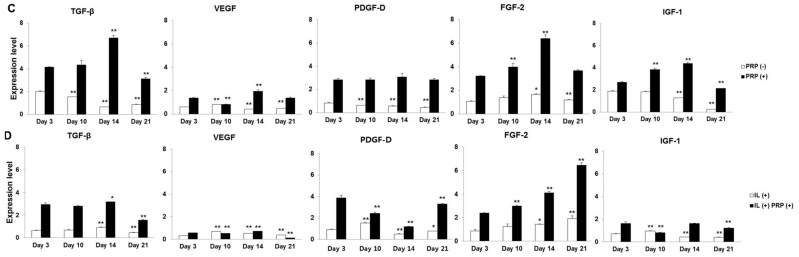
(**A**,**B**) Time sequential mRNA expression of each growth factor from meniscal cells with and without either PRP or IL-1α. The changes in the mRNA levels of TGF-β, VEGF, PDGF-D, and FGF-2 showed similar patterns between PRP(−) and PRP(+), as well as between IL-treated groups; (**C**,**D**) Time sequential protein expression of each growth factor from culture media of meniscal cells with and without either PRP or IL-1α. Protein levels of growth factors were higher in PRP(+) and IL(+)PRP(+)than in the other groups. * *p* < 0.05; ** *p* < 0.01 *versus* Day 3.

Changes in the mRNA levels of TGF-β, PDGF-D, and fibroblast growth factor-2 (FGF-2) were similar between PRP(−) and PRP(+). IGF-1 expression decreased in a time-dependent manner in PRP(+) (Figure 4A). The relative gene expression ratio of VEGF was higher (approximately 2–3-fold) in PRP(+) and IL(+)PRP(+) for the entire experimental period (Figure 4A,B). Gene expression levels of TGF-β, VEGF, PDGF-D, and FGF-2 had a similar pattern in both IL(+) and IL(+)PRP(+). Protein levels of general growth factors were higher in PRP(+) and IL(+)PRP(+)than in the other groups(Figure 4C,D).

#### 2.1.6. Apoptosis Evaluation of the Effects of PRP on Meniscal Cells

**Figure 5:**
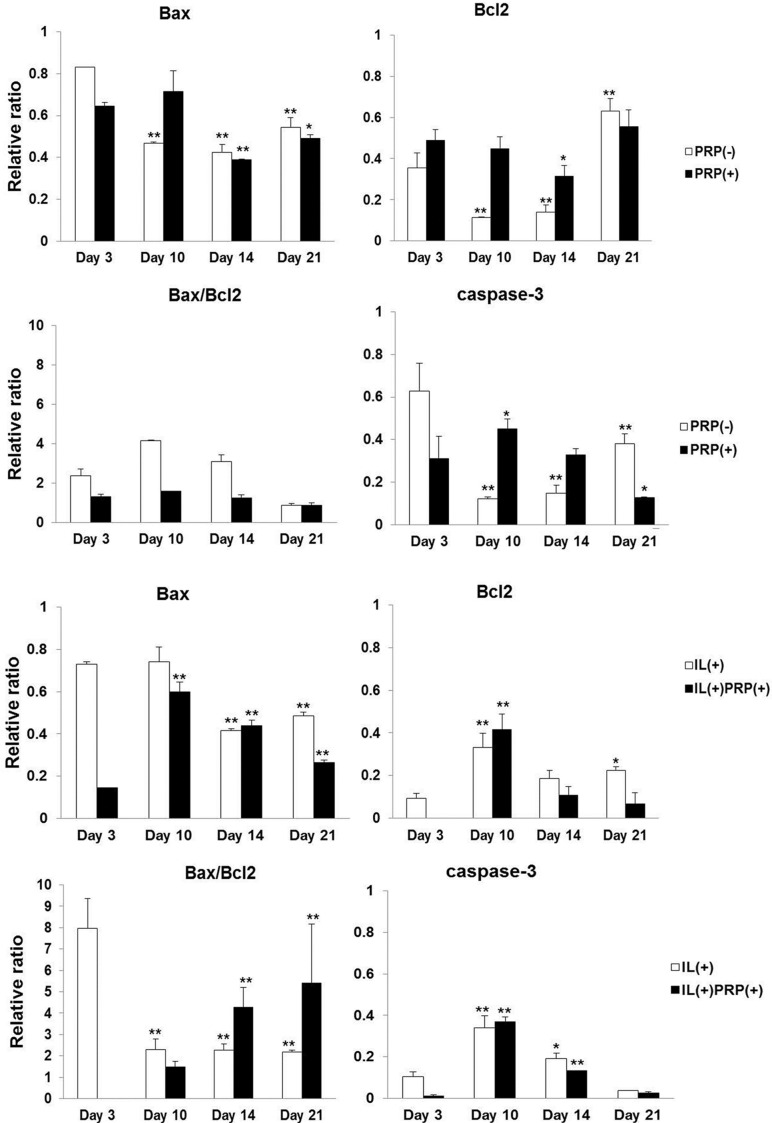
Effects of PRP on apoptosis in meniscal cells. The PRP(+) had no change in the ratio of Bax to Bcl2 mRNA in all examined periods, but the value of Bax/Bcl2 increased significantly in a time-dependent manner in IL(+)PRP(+). * *p* < 0.05; ** *p* < 0.01 *versus* Day 3.

The mRNA expression of caspase-3 decreased in a time-dependent manner in PRP with/without IL beginning at Day 3. Normal meniscal cells, PRP(−),exhibited gradual apoptosis in cultures, but PRP-treated cells did not exhibit significant changes in Bax/Bcl2 expression. In addition, the IL(+) group revealed a marked increase in Bax/Bcl2 on Day 3, which decreased four-fold afterwards and remained at that level for the remainder of the experimental period. The IL(+)PRP(+) showed sharp time-dependent increases in Bax/Bcl2, indicating the acceleration of apoptosis by PRP treatment (Figure 5). 

### 2.2. In Vivo

#### Histopathological Changes in Meniscal Wounds after PRP Implantation

**Figure 6:**
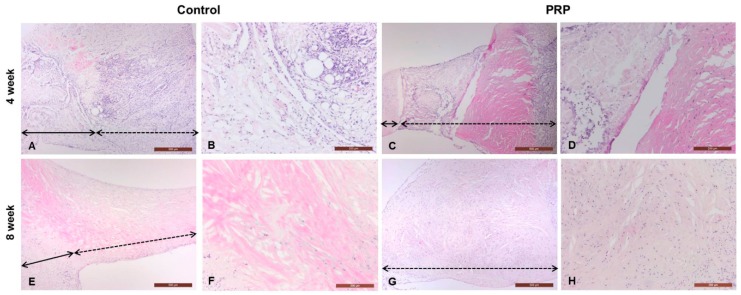
Histopathological analysis of full-thickness defects of the meniscus. The defective lesions of control and PRP-treated groups were completely replaced by fibrous tissue, instead of meniscal cartilage, at four and eight weeks. PRP-treated lesions were relatively thickened with hypercellularity of fibroblasts when compared to the control (black full line arrow: repair site, black dotted line arrow: non-defective site) H & E, magnifications 40× and 100×. **A** 4wk (40×)-Control; **B** 4wk (100×)-Control; **C** 4wk (40×)-PRP; **D** 4wk (100×)-PRP; **E** 8wk (40×)-Control; **F** 8wk (100×)-Control; **G** 8wk (40×)-PRP; **H** 8wk (100×)-PRP.

All rabbits were freely mobile in their cages post-operation and did not show signs of post-operative infection. At four and eight weeks, all groups showed diffuse hypercellularity at the wound sites and there was no degeneration of articular cartilage of the femur or tibia. At four weeks, defective lesions of the controls were composed of connective tissue showing severe fibrillation or disruption (Figure 6A,B). The PRP group revealed a distinct border between normal and reparative tissues. The reparative region was mostly composed of eosinophilic fibrous connective tissue (Figure 6C,D). At eight weeks, the menisci of controls was completely replaced by fibrous tissue, without meniscal cartilage formation (Figure 6E,F). PRP-treated lesions were also occupied by fibrous tissue, and the reparative tissue was thicker than that of the control (Figure 6G,H). The control and PRP groups demonstrated moderate fibrillation or markedly undulating lesions at eight weeks.

## 3. Discussion

Previous studies have shown conflicting results regarding the role of PRP in the regeneration of meniscal chondrocytes, including both positive [29,38,39] and negative [32,40] effects. Ishida *et al.* investigated whether PRP enhances meniscal tissue regeneration *in vivo*. They created 1.5-mm-diameter full-thickness defects in the avascular regions of rabbit menisci. The defects were filled with gelatin hydrogel (GH) with or without PRP. After four weeks of the implantation, fibrous tissues were abundant in the GH and PRP groups compared to the GH group [31]. These results were similar to our results. PRP-treated meniscal lesions were replaced by fibrous tissues, instead of normal meniscal cartilage, at four and eight weeks. In addition, in our *in vitro* study, the roles of PRP in the normal meniscus and the inflammatory meniscus were clarified, and changes in proteoglycans and collagens, as well as differences in the relative expression of growth factors in response to PRP treatment of meniscal cells were characterized. PRP was used as an autologous source of growth factors for the development of meniscal cells and improvement of meniscal injury by IL-1α. We focused on PRP-containing growth factors that were compared to a preparation from individual animals, and on the temporal and sequential changes related to the biochemical transition of meniscal cells.

According to previous reports, the levels of growth factors in humans do not differ with respect to age or sex [36,41]. Before exploring the application of PRP to meniscal cells, we compared growth factors in PRP derived from individual rabbits according to age and/or weight. Semi-quantitative RT-PCR analysis of PRPs from Y group and O group rabbits revealed that the Y group generally displayed higher expression of each growth factor, with the exception of IGF-1. However, protein levels of growth factors were higher in the O group than the Y group. Similar to observations in humans, growth factor of NZW rabbit have no relationship with aging. Accordingly, we used PRP derived from old, skeletally mature, rabbits in the subsequent experiments.

Cellular viability was influenced not only by concentration, but also by time sequence in cultures using a fixed number of PRP platelets (4 × 10^6^ platelets/μL) in the primary isolated meniscal cells from the avascular region of skeletally mature NZW rabbits. An MTT assay also revealed that 10% PRP result in maximum viability of meniscal cells. Therefore, we evaluated the biochemical changes of meniscal cells in normal cultures. IL-1α was used to mimic injury in cultures, as well as to induce injury in an *in vivo* model. Generally, IL-1α is highly expressed in OA in various regions, including the joint cartilage, synovium, and meniscus [10,42,43]. Indeed, acute exposure of meniscal tissue to IL-1α for just 1–3 days is sufficient to suppress tissue repair for up to four weeks [22]. IL-1α increases the expression and activity of MMPs in meniscal cells. Among them, the collagenase MMP-13 and the gelatinase MMP-9 are both associated with aggrecanase activity [44,45,46]. The MMPs are constrained by four forms of tissue inhibitors of MMPs (TIMPs), of which TIMP-1 and TIMP-2 are the most important MMP inhibitors. In addition to the inhibition of a wide spectrum of MMPs, TIMP-2 shows various biological functions important for hyaline cartilage [24,44,45,46]. In the present study, PRP stimulated the gradual loss of the major proteoglycan, aggrecan, in meniscal cells throughout the entire experimental period. In addition, type II collagen mRNA was markedly decreased by approximately 50% of the peak expression ratio at Day 14. The downregulation of type II collagen was associated with MMP-3, MMP-9, and MMP-13 responses to PRP(+). In sum, aggrecan and type II collagen in PRP(+) were altered, accompanied by changes in MMP-1 and MMP-13. Type I collagen and biglycan expression levels were lower in PRP(+) than PRP(−). IL-1α-induced inflammation acted as a surrogate of *in vivo* meniscus injury. IL(+) and IL(+)PRP(+) resulted in the loss of aggrecan and type II collagen. These results indicated that meniscal inflammation stimulates remarkable proteoglycanolysis, and further indicate the catabolic role of PRP in meniscal injury, distinct from normal meniscal cells as well as PRP-treated joint cartilage [9,47,48]. These results were also confirmed by the high and long-term expression of MMP-1 and MMP-3 in the IL(+)PRP(+) compared to IL(+). PRP(+) generally expressed high levels of MMPs and TIMPs in meniscal cells throughout the experimental period. The PRP(+) exhibited relatively low levels of Bax/Bcl2 during the culture period and these levels were not different from those in the PRP(−). There was noticeable proteoglycanolysis and a decrease of type II collagen in PRP(+), which was related to caspase-3 expression. For IL-1α-induced inflammation, the PRP-treated group had a marked increase in Bax/Bcl2, indicating a time-dependent acceleration of apoptosis, whereas the IL(+) revealed high levels of Bax/Bcl2 only at Day 3. PRP treatment in normal meniscal cartilage did not lead to significant changes in apoptosis, but rather to a remarkable proteoglycanolysis with imbalance between type II and type I collagens. Furthermore, PRP treatment in the inflammatory meniscus causes severe proteoglycanolysis and a loss of type II collagen with increasing apoptosis, which leads to fibrous lesions instead of meniscal cartilage recovery by laceration, rupture, OA and synovitis.

According to previous studies, growth factors in PRP, especially TGF-β and PDGF-D, are helpful in repairing meniscal injury repair [19,25,30,31,32]. In the present study, TGF-β, PDGF-D, FGF-2, and IGF-1 were upregulated in PRP(+) during the experimental period. These growth factors were also highly expressed in IL(+)PRP(+). Nonetheless, we observed proteoglycanolysis and collagenolysis. In our *in vivo* study, PRP-treated meniscal lesions were replaced by fibrous tissues instead of normal meniscal cartilage. Almost all defects consisted of stromal tissues after eight weeks of the healing process which were uneven between the control and PRP-treated rabbits, despite the limitations in the examined models between the *in vivo* full-thickness wound of the meniscus and the *in vitro* primary isolated meniscal cells from the inner avascular region. However, the reparative tissues also revealed hypertrophic fibrous stroma, instead of the meniscal cartilage, in PRP-treated rabbits, similar to the *in vitro* results.

In conclusion, our results show that the upregulation of most growth factors was not related to chondrogenesis. As a consequence of the high expression of MMPs, aggrecan and/or type II collagen expression levels were significantly downregulated in PRP(+) and IL(+)PRP(+). Furthermore, we found that PRP primarily affects the dedifferentiation of meniscal cells by increasing type I collagen and decreasing aggrecan in IL-1α-treated meniscal cells. Thus, in clinical trials, local administration of PRP would lead to side effects for meniscal repair, owing to proteoglycanolysis via the upregulation of catabolic molecules and the an increase in type I collagen, resulting in fibrous tissue formation, rather than meniscal cartilage.

## 4. Experimental Section

### 4.1. Animals

Fourteen male NZW rabbits weighing 1.5–2.6 kg (Samtako, Daejeon, Korea) were used to isolate meniscal cells and to prepare PRP. Sixteen male NZW rabbits weighing 3.0 kg were used as meniscal defect animal models. All experimental protocols were approved by the Institutional Animal Care and Use Committee of Yeungnam University (2011).

### 4.2. In Vitro

#### 4.2.1. PRP Preparation

RT-PCR and ELISA were performed for individual growth factors in PRP prepared from young rabbits (Y, age 4 weeks, weight 1.5–1.7 kg) and old rabbits (O, age 16 weeks, weight 2.3–2.6 kg). Each group contained seven rabbits (R1–R7) that were evaluated individually.

PRP was prepared as previously described [35,36,37,49]. Briefly, 40 mL of blood were obtained from rabbits and mixed with the anti-coagulant solution acid citrate dextrose A. The mixture was centrifuged at 1500× *g* for 10 min to separate the red blood cells from the platelet-containing plasma. The collected supernatant was then centrifuged at 3000× *g* for 10 min, and the pelleted platelets were collected. The pellet was resuspended in 4 mL of plasma. Platelet counts were analyzed using an ADVOA 2120 Automatic Counter (Bayer Diagnostic GmbH, Leverkusen, Germany).

#### 4.2.2. Enzyme-Linked Immunosorbent Assay (ELISA)

The PRP of each rabbit (Y and O) was tested for antibodies against growth factors using ELISA [50]. Briefly, 96-well plates were coated with diluted PRP at 4 °C overnight, after which they were washed three times with washing buffer. After blocking with 1% (*w*/*v*) non-fat dried milk (skim milk; Difco, BD Bioscience, Franklin Lakes, NJ, USA), the plates were incubated with the following antibodies for ELISA: monoclonal anti-VEGF; polyclonal rabbit anti- TGF-β, PDGF-D, fibroblast growth factor-2 (FGF-2); and polyclonal goat anti-insulin-like growth factor-1 (IGF-1) (Santa Cruz Biotechnology, Heidelberg, Germany, 1:200). After the final set of washes, 50 μL of fresh TMB substrate (Koma Biotech, Seoul, Korea) were added to each well and incubated. After sufficient color development, 50 μL of stop buffer (2 M H_2_SO_4_, Koma Biotech, Seoul, Korea) were added to the wells. Optical densities (at 450 nm) were measured using a Sunrise Microplate Reader (Tecan, Salzburg, Austria). All samples were run in triplicate.

#### 4.2.3. Isolation and Culturing of Meniscal Cells

Male, 16 week-old NZW rabbits weighing 2.3–2.6 kg were euthanized with an injection of tiletamine and zolazepam (Zoletil 50^®^ 0.2 mL/kg, intramuscular (IM); Virbac, Seoul, Korea), and the intact lateral and medial menisci were carefully removed from the knees. The menisci were then manually and enzymatically digested for 2 h with 2 mg/mL collagenase type II (*Clostridium histolyticum*; Gibco, Carlsbad, CA, USA) in phosphate-buffered saline (PBS; Gibco) at 37 °C. Free cells were passed through a sterile 70-μm nylon mesh (BD Biosciences, Bedford, MA, USA) to remove undigested fragments, and the isolated meniscal cells were then washed three times with Dulbecco’s Modified Eagle Medium (DMEM) supplemented with 10% fetal bovine serum (FBS) and 1% antibiotics (all from Gibco). A pellet of meniscal cells was obtained by centrifugation of the previously mentioned solution at 3000× *g* for 5 min, and the cells were then re-suspended in culture medium. Cells were counted using a hemocytometer, and viability was determined using the trypan blue exclusion test (Sigma-Aldrich, St. Louis, MO, USA). Following the confluent growth of meniscal cells, the medium was changed every 2–3 days. After three passages, the meniscal cells were used for the *in vitro* PRP treatment assays.

#### 4.2.4. Cell Viability

The effects of PRP on meniscal cells were determined using a viability assay based on MTT with a commercial kit (Roche Diagnostic, Basel, Switzerland). Briefly, meniscal cells were plated into 96-well plates (2 × 10^2^ cells/well) and were then cultured in 0%, 0.1%, 1%, 5%, 10%, and 20% PRP in DMEM supplemented with 1% FBS, for Days 1, 4, and 7 after a single PRP treatment. MTT solution was added to the cells for 4 h to allow the formation of a water-insoluble formazan dye. After solubilization using dimethylsulfoxide, the amount of formazan dye released was determined at 595 nm using a Sunrise Microplate Reader (Tecan, Zürich, Switzerland).

#### 4.2.5. RNA Extraction and Semi-Quantitative RT-PCR

The meniscal cells were incubated with and without 10% PRP or 3 ng/mL recombinant human (rh)IL-1α (R&D Systems, Minneapolis, MN, USA) and were then cultured in FBS-free DMEM for Days 3, 10, 14, and 21. Meniscal cells were divided into four groups: PRP(−), PRP(+), with IL-1α (IL(+)), and IL-1α and PRP (IL(+)PRP(+)). Total RNA was extracted from the PRP prepared from Y and O rabbits and cultured cells using RNAiso Plus (Takara, Shiga, Japan), following the manufacturer’s instructions. DNA concentration was determined using a NanoVue spectrophotometer (GE Healthcare, Freiburg, Germany). One microgram of total RNA was converted to complementary DNA by a reverse transcription reaction prior to the use of the PCR Premix Kit (Bioneer, Daejeon, Korea) in an Eppendorf Mastercycler^®^ (Eppendorf, Hamburg, Germany). Serial changes in the expression of the target genes were evaluated using semi-quantitative RT-PCR, with the glyceraldehyde 3-phosphate dehydrogenase (GAPDH) gene as a control. The ImageJ software (National Institutes of Health, Bethesda, MD, USA) was used to quantify the band intensity of the RT-PCR amplicons for primers. The primer sets used in the PCR are listed in Table 1. All samples were run in triplicate.

#### 4.2.6. Immunoblot Analysis

Culture media of meniscal cells were divided into four groups: PRP(−), PRP(+), IL(+) and IL(+)PRP(+). The culture media were mixed with RIPA buffer containing protease inhibitor cocktail tablets (Roche, Mannheim, Germany) [51,52,53]. Subsequently, the solution was centrifuged at 12,000× *g* for 10 min at 4 °C to obtain soluble protein in the supernatant. The protein concentration was determined using the Bradford method [54]. Proteins of interest were immunoprecipitated using 500 μg of total protein, following standard protocols [51,53]. Immunoprecipitated proteins were resolved using sodium dodecyl sulfate polyacrylamide gel electrophoresis, and were then transferred to polyvinylidene difluoride transfer membranes. After blocking with 3% bovine serum albumin (BSA; Sigma-Aldrich), the membranes were incubated with antibodies for immunoblot analysis: monoclonal mouse anti- β-actin, VEGF; and polyclonal rabbit anti- MMP-9, MMP-13, TIMP-1, TIMP-2, TGF-β, PDGF-D, and FGF-2; and polyclonal goat anti- MMP-1, MMP-3, and IGF-1 (Santa Cruz Biotechnology, 1:200). β-actin was used as a loading control in the immunoblot analyses. Specific binding was detected using the SuperSignal West Dura Extended Duration Substrate (PIERCE, Rockford, IL, USA), and the blots were exposed to medical X-ray film (Kodak, Tokyo, Japan). The images of protein bands were scanned, and band intensities were quantified using ImageJ software. All samples were run in triplicate.

**Table 1:** The primer sequences provided in the study.

Category	Primer	Gene Bank	Size (bp)	Sequence (5′-3′)
**Chondrogenesis**	**GAPDH**	DQ403051.1	304	TCA CCA TCT TCC AGG AGC GA
				CAC AAT GCC GAA GTG GTC GT
	**Aggrecan**	XM002723376.1	744	CTC ACC CCG AGA ATC AAA TG
				AGG AGG TTT CCG CCG CAG TT
	**Type I Collagen**	XM 002713800.1	310	CGC GAT GGT CAG CCT GGA CA
				CCG GGA GGG CCA GCA GGA CC
	**Type II Collagen**	D 83228.1	370	GAC CCC ATG CAG TAC ATG
				AGC CGC CAT TGA TGG TCT CC
	**Biglycan**	XM 002722633.1	386	CAC TGC CAC CTG CGG GTT GT
				TCT AGG GGG TTC CCG CCC ATC
**Catabolic molecule**	**MMP-1**	NM 001171139.1	553	GCA ACC CAG GTG TGG AGT GCC
				TGG GCC TAC TGG CTG ACT GGG
	**MMP-3**	NM 001082280.1	339	CTG GAG GTT TGA TGA GAA GA
				CAG TTC ATG CTC GAG ATT CC
	**MMP-9**	NM 001082203.1	519	GAC GGC AAG CCC TGC GAG TT
				TGT GGT GGT GGC TGG AGG CT
	**MMP-13**	NM 001082037.1	328	GCA GCA GTC TCC AGG CAC GG
				TCA GGG ACC CCG CAT CTC GG
**Anabolic molecule**	**TIMP-1**	NM 001082232.2	326	GCA ACT CCG ACC TTG TCA TC
				AGC GTA GGT CTT GGT GAA GC
	**TIMP-2**	XM 002723776.1	460	AAC GGA GTC TGG TGG TGC ATT CC
				CTT GGC CTG GTG CCC GTT GAT
**Growth factor**	**TGF-β**	AB 020217.1	231	GCA AGG ACC TGG GCT GGA A
				AGT AAC ACG ATG GGC AGT GGC
	**VEGF**	XM 002714697.1	100	CGC AGC TAC TGC CAG CCG AT
				GCA CCA GAG GCA CGC AGG AA
	**PDGF-D**	XM 002708534.1	125	TGC ACC GGC TCA TCC TCG TCT A
				GTC ATC TCG CCG GAG ATT GGC GTT G
	**FGF-2**	XM 002717238.1	278	GGA GAA GAG CGA CCC ACA CAT CA
				TAG CCT TCT GCC CAG GTC CTG TT
	**IGF-1**	NM 001082026.1	194	TCT GCG GTG CTG AGC TGG TG
				TGC CTT TGC CGG CTT GAG GG
**Apoptosis**	**Bcl2**	DQ 529234.1	233	GTG GGA TAC TGG AGA TGA AGA
				GAC GGT AGC GAC GAG AGA
	**Bax**	XM 002723697.1	400	CCA AGA AGC TGA GCG AGT G
				TTC CAG ATG GTG AGT GAG G
	**caspase-3**	NM 001082280.1	489	CAA TGG ACT CTG GGA AAT
				GCA AGC CTG AAT AAT GA

### 4.3. In Vivo

#### 4.3.1. Meniscal Defect for PRP Application in Rabbits

A full-thickness circular defect was created on the menisci of skeletally mature male NZW rabbits, with a mean weight of 3.0 kg. Surgical procedures were performed as previously described [9,55,56,57]. After general anesthesia was induced by tiletamine and zolazepam (Zoletil 50^®^ 0.2 mL/kg, IM), each rabbit was placed in a supine position and surgery was performed bilaterally on the knees. A medial parapatellar approach was used to expose the knee joint, and the patella was averted and flexed to the maximum. A 2 mm-diameter full-thickness circular defect was created in the anterior portion of the inner two-thirds of the avascular zone of the medial meniscus by using a dermal biopsy punch (Miltex, Rietheim-Weilheim, Germany). The animals were then divided into two groups, *i.e.*, control and PRP groups. In the control group (*n* = 8), defects were filled with fibrin glue (Green Cross, Seoul, Korea). In the PRP group (*n* = 8), defects were filled with 10% PRP and were then covered with fibrin glue. The joint capsule and skin were sutured as separate layers in all animals. After surgery, all rabbits were allowed to move freely without joint immobilization. At 4 and 8 weeks post-operation, the rabbits were periodically sacrificed. The meniscus was collected from the knee joint and was evaluated histochemically.

#### 4.3.2. Histopathological Analysis

The menisci were fixed in 10% neutral-buffered formalin and were embedded in paraffin. Each meniscus was cut into 4-μm-thick sections. For the histopathological analysis, the sections were stained with hematoxylin and eosin (H & E). The reparative tissue was evaluated by microscopic examination. In addition, for the histological analysis, the quality of the surface area, integration, cellularity, and cell morphology was evaluated in reparative tissue from three random fields of view (each group (*n* = 4)) using a microscope.

### 4.4. Statistical Analysis

Statistical analysis was performed using Prism 4.02 (GraphPad Software, San Diego, CA, USA). Multiple comparisons were analyzed using one-way analysis of variance (ANOVA) followed by a Dunnett’s post-hoc test, and results are expressed as mean values and their respective standard deviations. The data were considered to be significantly different at *p* < 0.05, *p* < 0.01, or *p* < 0.001.

## References

[1] Kuettner K.E., Cole A.A. (2005). Cartilage degeneration in different human joints. Osteoarthr. Cartil..

[2] Almarza A.J., Athanasiou K.A. (2004). Seeding techniques and scaffolding choice for tissue engineering of the temporomandibular joint disk. Tissue Eng..

[3] Aigner T., Stöve J. (2003). Collagens—Major component of the physiological cartilage matrix, major target of cartilage degeneration, major tool in cartilage repair. Adv. Drug Deliv. Rev..

[4] Verdonk P.C., Forsyth R.G., Wang J., Almqvist K.F., Verdonk R., Veys E.M., Verbruggen G. (2005). Characterisation of human knee meniscus cell phenotype. Osteoarthr. Cartil..

[5] McDevitt C.A., Webber R.J. (1990). The ultrastructure and biochemistry of meniscal cartilage. Clin. Orthop. Relat. Res..

[6] Collier S., Ghosh P. (1995). Effects of transforming growth factor beta on proteoglycan synthesis by cell and explant cultures derived from the knee joint meniscus. Osteoarthr. Cartil..

[7] Makris E.A., Hadidi P., Athanasiou K.A. (2011). The knee meniscus: Structure-function, pathophysiology, current repair techniques, and prospects for regeneration. Biomaterials.

[8] Mauck R.L., Martinez-Diaz G.J., Yuan X., Tuan R.S. (2007). Regional multilineage differentiation potential of meniscal fibrochondrocytes: Implications for meniscus repair. Anat. Rec..

[9] Higashioka M.M., Chen J.A., Hu J.C., Athanasiou K.A. (2014). Building an anisotropic meniscus with zonal variations. Tissue Eng. A.

[10] Horie M., Driscoll M.D., Sampson H.W., Sekiya I., Caroom C.T., Prockop D.J., Thomas D.B. (2010). Implantation of allogenic synovial stem cells promotes meniscal regeneration in a rabbit meniscal defect model. J. Bone Jt. Surg. Am..

[11] Scotti C., Hirschmann M.T., Antinolfi P., Martin I., Peretti G.M. (2013). Meniscus repair and regeneration: Review on current methods and research potential. Eur. Cell Mater..

[12] Furman B.D., Kimmerling K.A., Zura R.D., Reilly R.M., Zlowodzki M.P., Huebner J.L., Kraus V.B., Guilak F., Olson S.A. (2015). Articular ankle fracture results in increased synovitis, synovial macrophage infiltration, and synovial fluid concentrations of inflammatory cytokines and chemokines. Arthritis Rheumatol..

[13] Moyer H.R., Wang Y., Farooque T., Wick T., Singh K.A., Xie L., Guldberg R.E., Williams J.K., Boyan B.D., Schwartz Z. (2010). A new animal model for assessing cartilage repair and regeneration at a nonarticular site. Tissue Eng. A.

[14] Riera K.M., Rothfusz N.E., Wilusz R.E., Weinberg J.B., Guilak F., McNulty A.L. (2011). Interleukin-1, tumor necrosis factor-α, and transforming growth factor-β 1 and integrative meniscal repair: Influences on meniscal cell proliferation and migration. Arthritis Res. Ther..

[15] Lotz M. (2001). Cytokines in cartilage injury and repair. Clin. Orthop. Relat. Res..

[16] Schlaak J.F., Pfers I., Meyer ZumBüschenfelde K.H., Märker-Hermann E. (1996). Different cytokine profiles in the synovial fluid of patients with osteoarthritis, rheumatoid arthritis and seronegative spondylarthropathies. Clin. Exp. Rheumatol..

[17] Ferretti M., Madhavan S., Deschner J., Rath-Deschner B., Wypasek E., Agarwal S. (2006). Dynamic biophysical strain modulates proinflammatory gene induction in meniscal fibrochondrocytes. Am. J. Physiol. Cell Physiol..

[18] Upton M.L., Chen J., Setton L.A. (2006). Region-specific constitutive gene expression in the adult porcine meniscus. J. Orthop. Res..

[19] Ruiz Ibán M.Á., Comellas Melero N., Martinez-Botas J., Ortiz A., Diaz Heredia J. (2014). Growth factor expression after lesion creation in the avascular zone of the meniscus: A quantitative PCR study in rabbits. Arthroscopy.

[20] Argentieri E.C., Sturnick D.R., DeSarno M.J., Gardner-Morse M.G., Slauterbeck J.R., Johnson R.J., Beynnon B.D. (2014). Changes to the articular cartilage thickness profile of the tibia following anterior cruciate ligament injury. Osteoarthr. Cartil..

[21] Aghaloo T.L., Moy P.K., Freymiller E.G. (2002). Investigation of platelet-rich plasma in rabbit cranial defects: A pilot study. J. Oral Maxillofac. Surg..

[22] Kanthan S.R., Kavitha G., Addi S., Choon D.S., Kamarul T. (2011). Platelet-rich plasma (PRP) enhances bone healing in non-united critical-sized defects: A preliminary study involving rabbit models. Injury.

[23] Eppley B.L., Woodell J.E., Higgins J. (2004). Platelet quantification and growth factor analysis from platelet-rich plasma: Implications for wound healing. Plast. Reconstr. Surg..

[24] Ionescu L.C., Lee G.C., Huang K.L., Mauck R.L. (2012). Growth factor supplementation improves native and engineered meniscus repair *in vitro*. Acta Biomater..

[25] Izal I., Ripalda P., Acosta C.A., Forriol F. (2008). *In vitro* healing of avascular meniscal injuries with fresh and frozen plugs treated with TGF-β1 and IGF-1 in sheep. Int. J. Clin. Exp. Pathol..

[26] Tumia N.S., Johnstone A.J. (2009). Platelet derived growth factor-AB enhances knee meniscal cell activity *in vitro*. Knee.

[27] Mishra A., Tummala P., King A., Lee B., Kraus M., Tse V., Jacobs C.R. (2009). Buffered platelet-rich plasma enhances mesenchymal stem cell proliferation and chondrogenic differentiation. Tissue Eng. C Methods.

[28] Lee H.R., Park K.M., Joung Y.K., Park K.D., Do S.H. (2012). Platelet-rich plasma loaded *in situ*-formed hydrogel enhances hyaline cartilage regeneration by CB1 upregulation. J. Biomed. Mater. Res. A.

[29] Lee H.R., Park K.M., Joung Y.K., Park K.D., Do S.H. (2012). Platelet-rich plasma loaded hydrogel scaffold enhances chondrogenic differentiation and maturation with up-regulation of CB1 and CB2. J. Control. Release.

[30] Buma P., Ramrattan N.N., van Tienen T.G., Veth R.P. (2004). Tissue engineering of the meniscus. Biomaterials.

[31] Ishida K., Kuroda R., Miwa M., Tabata Y., Hokugo A., Kawamoto T., Sasaki K., Doita M., Kurosaka M. (2007). The regenerative effects of platelet-rich plasma on meniscal cells *in vitro* and its *in vivo* application with biodegradable gelatin hydrogel. Tissue Eng..

[32] Zellner J., Taeger C.D., Schaffer M., Roldan J.C., Loibl M., Mueller M.B., Berner A., Krutsch W., Huber M.K., Kujat R. (2014). Are applied growth factors able to mimic the positive effects of mesenchymal stem cells on the regeneration of meniscus in the avascular zone?. Biomed. Res. Int..

[33] Fennis J.P., Stoelinga P.J., Merkx M.A., Jansen J.A. (2009). Reconstruction of the mandible with a poly(d,l-lactide) scaffold, autogenous corticocancellous bone graft, and autogenous platelet-rich plasma: An animal experiment. Tissue Eng..

[34] Yamada Y., Ueda M., Naiki T., Takahashi M., Hata K., Nagasaka T. (2004). Autogenous injectable bone for regeneration with mesenchymal stem cells and platelet-rich plasma: Tissue-engineered bone regeneration. Tissue Eng..

[35] Van den Dolder J., Mooren R., Vloon A.P., Stoelinga P.J., Jansen J.A. (2006). Platelet-rich plasma: Quantification of growth factor levels and the effect on growth and differentiation of rat bone marrow cells. Tissue Eng..

[36] Cho H.S., Song I.H., Park S.Y., Sung M.C., Ahn M.W., Song K.E. (2011). Individual variation in growth factor concentrations in platelet-rich plasma and its influence on human mesenchymal stem cells. Korean J. Lab. Med..

[37] Park S.I., Lee H.R., Kim S., Ahn M.W., Do S.H. (2012). Time-sequential modulation in expression of growth factors from platelet-rich plasma (PRP) on the chondrocyte cultures. Mol. Cell. Biochem..

[38] Chen W.H., Lo W.C., Hsu W.C., Wei H.J., Liu H.Y., Lee C.H., Tina Chen S.Y., Shieh Y.H., Williams D.F., Deng W.P. (2014). Synergistic anabolic actions of hyaluronic acid and platelet-rich plasma on cartilage regeneration in osteoarthritis therapy. Biomaterials.

[39] Narita A., Takahara M., Sato D., Ogino T., Fukushima S., Kimura Y., Tabata Y. (2012). Biodegradable gelatin hydrogels incorporating fibroblast growth factor 2 promote healing of horizontal tears in rabbit meniscus. Arthroscopy.

[40] Griffin J.W., Hadeed M.M., Werner B.C., Diduch D.R., Carson E.W., Miller M.D. (2015). Platelet-rich plasma in meniscal repair: Does augmentation improve surgical outcomes?. Clin. Orthop. Relat. Res..

[41] Dimauro I., Grasso L., Fittipaldi S., Fantini C., Mercatelli N., Racca S., Geuna S., di Gianfrancesco A., Caporossi D., Pigozzi F. (2014). Platelet-rich plasma and skeletal muscle healing: A molecular analysis of the early phases of the regeneration process in an experimental animal model. PLoS ONE.

[42] McNulty A.L., Weinberg J.B., Guilak F. (2009). Inhibition of matrix metalloproteinases enhances *in vitro* repair of the meniscus. Clin. Orthop. Relat. Res..

[43] Lemke A.K., Sandy J.D., Voigt H., Dreier R., Lee J.H., Grodzinsky A.J., Mentlein R., Fay J., Schünke M., Kurz B. (2010). Interleukin-1α treatment of meniscal explants stimulates the production and release of aggrecanase-generated, GAG-substituted aggrecan products and also the release of pre-formed, aggrecanase-generated G1 and m-calpain-generated G1–G2. Cell Tissue Res..

[44] Zwierzchowski T.J., Stasikowska-Kanicka O., Danilewicz M., Fabiś J. (2012). Assessment of apoptosis and MMP-1, MMP-3 and TIMP-2 expression in tibial hyaline cartilage after viable medial meniscus transplantation in the rabbit. Arch. Med. Sci..

[45] Groma G., Xin W., Grskovic I., Niehoff A., Brachvogel B., Paulsson M., Zaucke F. (2012). Abnormal bone quality in cartilage oligomeric matrix protein and matrilin 3 double-deficient mice caused by increased tissue inhibitor of metalloproteinases 3 deposition and delayed aggrecan degradation. Arthritis Rheum..

[46] Toegel S., Wu S.Q., Otero M., Goldring M.B., Leelapornpisid P., Chiari C., Kolb A., Unger F.M., Windhager R., Viernstein H. (2012). Caesalpiniasappan extract inhibits IL1β-mediated overexpression of matrix metalloproteinases in human chondrocytes. Genes Nutr..

[47] Rai M.F., Patra D., Sandell L.J., Brophy R.H. (2013). Transcriptome analysis of injured human meniscus reveals a distinct phenotype of meniscus degeneration with aging. Arthritis Rheum..

[48] Eppley B.L., Pietrzak W.S., Blanton M. (2006). Platelet-rich plasma: A review of biology and applications in plastic surgery. Plast. Reconstr. Surg..

[49] Rasheed Z., Akhtar N., Khan A., Khan K.A., Haqqi T.M. (2010). Butrin, isobutrin, and butein from medicinal plant *Butea monosperma* selectively inhibit nuclear factor-κB in activated human mast cells: Suppression of tumor necrosis factor-α, interleukin (IL)-6, and IL-8. J. Pharmacol. Exp. Ther..

[50] Hamilton B., Tol J.L., Knez W., Chalabi H. (2013). Exercise and the platelet activator calcium chloride both influence the growth factor content of platelet-rich plasma (PRP): Overlooked biochemical factors that could influence PRP treatment. Br. J. Sports Med..

[51] Abrams G.D., Hussey K.E., Harris J.D., Cole B.J. (2014). Clinical results of combined meniscus and femoral osteochondral allograft transplantation: Minimum 2-year follow-up. Arthroscopy.

[52] Kim Y.H., Jung J.C. (2012). Suppression of tunicamycin-induced CD44v6 ectodomain shedding and apoptosis is correlated with temporal expression patterns of active ADAM10, MMP-9 and MMP-13 proteins in Caki-2 renal carcinoma cells. Oncol. Rep..

[53] Guo J., Jie W., Shen Z., Li M., Lan Y., Kong Y., Guo S., Li T., Zheng S. (2014). SCF increases cardiac stem cell migration through PI3K/AKT and MMP-2/-9 signaling. Int. J. Mol. Med..

[54] Bradford M.M. (1976). A rapid and sensitive method for the quantitation of microgram quantities of protein utilizing the principle of protein-dye binding. Anal. Biochem..

[55] Nagae M., Ikeda T., Mikami Y., Hase H., Ozawa H., Matsuda K., Sakamoto H., Tabata Y., Kawata M., Kubo T. (2007). Intervertebral disc regeneration using platelet-rich plasma and giodegradable gelatin hydrogel microspheres. Tissue Eng..

[56] Marx R.E. (2004). Platelet-rich plasma: Evidence to support its use. J. Oral Maxillofac. Surg..

[57] Pauli C., Grogan S.P., Patil S., Otsuki S., Hasegawa A., Koziol J., Lotz M.K., D’Lima D.D. (2011). Macroscopic and histopathologic analysis of human knee menisci in aging and osteoarthritis. Osteoarthr. Cartil..

